# The Indictment and the Defense

**Published:** 1888-11

**Authors:** 


### *THE INDICTMENT AND THE DEFENSE.*

The case of the late Emperor Frederick has called forth a public controversy between the German surgeons and Sir Morell Mackenzie, who attended him during his fatal illness, and this war of words is anything but creditable to scientific men. Sir Morell is arraigned by his German associates in the case, and he is charged with making an erroneous diagnosis, with discouraging the most proper yet radical treatment, with being bungling in his manipulations, and unskilful in the selection and application of remedies, and with discourteous and unprofessional methods. Dr. Mackenzie defends himself by denying the accusations of his German confréres, by making counter-charges of unprofessional conduct, of improper treatment, and of questionable diagnosis. He also fortifies himself with argument and statistics. Much of the controversy resolves itself into questions of veracity, professional knowledge, and skill in therapeutics. There will be differences of opinion as to who is right, some taking one position, others the opposite.

It is to be hoped, however, that, out of this passionate contention, some good may yet come. The profession will be drawn to a more careful consideration of laryngeal disease, the various kinds of treatment will be more thoroughly reviewed and discussed, and a more trustworthy consensus of therapeutic opinion established. Especially will the facts and arguments which will be incidentally brought forward help to answer two important questions, viz.: 1st. In intra-laryngeal growths not eradicable by the mouth, is early thyrotomy and thorough removal of such growths the proper treatment? 2d. Is unilateral or complete excision of the larynx in supposed malignant intra-laryngeal disease justifiable at any period of the malady, and if so, when? Another question which will most prominently come forward and seek reply, will be: May a benign growth be transformed into a malignant one?

These questions are all of scientific value, and out of the heat of this unseemly controversy there may come some light which will aid in answering them.

---

Dr. J. O. Roe, of Rochester, in a recent interview with the reporter of the *Post-Express*, gave a most excellent, full, and frank opinion of the controversy between Sir Morell Mackenzie and the German physicians of the late Emperor Frederick III. The long and intimate personal friendship existing between Sir Morell and Dr. Roe, together with the fact that the latter has so recently visited the former, adds peculiar interest and weight to the opinions expressed.

